# CATA: a comprehensive chromatin accessibility database for cancer

**DOI:** 10.1093/database/baab085

**Published:** 2022-01-17

**Authors:** Jianyuan Zhou, Yanshang Li, Haotian Cao, Min Yang, Lingyu Chu, Taisong Li, Zhengmin Yu, Rui Yu, Bo Qiu, Qiuyu Wang, Xuecang Li, Jianjun Xie

**Affiliations:** Central Laboratory of Molecular Biology, Medical College of Jiaying University, 146 Huangtang Road, Meizhou 514031, China; Shandong Provincial Key Laboratory of Radiation Oncology, Cancer Research Center, Shandong Cancer Hospital and Institute, Shandong First Medical University, and Shandong Academy of Medical Sciences, 440 Jiyan Road, Jinan 250000, China; Central Laboratory of Molecular Biology, Medical College of Jiaying University, 146 Huangtang Road, Meizhou 514031, China; Department of Biochemistry and Molecular Biology, Shantou University Medical College, 22 Xinling Road, Shantou 515041, China; Department of Oral & Maxillofacial Surgery, Sun Yat-Sen Memorial Hospital, Sun Yat-Sen University, 107 Yanjiang Xilu, Guangzhou 510000, China; Department of Biochemistry and Molecular Biology, Shantou University Medical College, 22 Xinling Road, Shantou 515041, China; Department of Biochemistry and Molecular Biology, Shantou University Medical College, 22 Xinling Road, Shantou 515041, China; Department of Biochemistry and Molecular Biology, Shantou University Medical College, 22 Xinling Road, Shantou 515041, China; School of Medical Informatics, Harbin Medical University, Daqing Campus, Daqing 163319, China; School of Medical Informatics, Harbin Medical University, Daqing Campus, Daqing 163319, China; Central Laboratory of Molecular Biology, Medical College of Jiaying University, 146 Huangtang Road, Meizhou 514031, China; School of Medical Informatics, Harbin Medical University, Daqing Campus, Daqing 163319, China; School of Medical Informatics, Harbin Medical University, Daqing Campus, Daqing 163319, China; Department of Biochemistry and Molecular Biology, Shantou University Medical College, 22 Xinling Road, Shantou 515041, China

## Abstract

Accessible chromatin refers to the active regions of a chromosome that are bound by many transcription factors (TFs). Changes in chromatin accessibility play a critical role in tumorigenesis. With the emergence of novel methods like Assay for Transposase-accessible Chromatin Sequencing, a sequencing method that maps chromatin-accessible regions (CARs) and enables the computational analysis of TF binding at chromatin-accessible sites, the regulatory landscape in cancer can be dissected. Herein, we developed a comprehensive cancer chromatin accessibility database named CATA, which aims to provide available resources of cancer CARs and to annotate their potential roles in the regulation of genes in a cancer type-specific manner. In this version, CATA stores 2 991 163 CARs from 23 cancer types, binding information of 1398 TFs within the CARs, and provides multiple annotations about these regions, including common single nucleotide polymorphisms (SNPs), risk SNPs, copy number variation, somatic mutations, motif changes, expression quantitative trait loci, methylation and CRISPR/Cas9 target loci. Moreover, CATA supports cancer survival analysis of the CAR-associated genes and provides detailed clinical information of the tumor samples.

**Database URL**: CATA is available at http://www.xiejjlab.bio/cata/.

## Introduction

Accessible chromatin is a hallmark of an active DNA regulatory element (1). The identification of chromatin accessibility makes it possible to assess the regulatory landscape for human cancers because active chromatin contains a variety of gene regulatory information (2, 3). Chromatin accessibility analysis has been shown to be able to identify transcription factor (TF) binding sites and regulatory elements, such as achaete-scute complex-like 1 gene (4) and ARID1A (5). Also, Chromatin-accessible regions (CARs) in different tumors are highly specific. For example, there are many specific CARs and CARs-related genes that are closely related to breast cancer (1), whereas they are rarely present in other cancer types. Cancers also share some common open regions of chromatin. For instance, the promoter of programmed cell death ligand 1 (PDL1), a tumor marker widely existing in cancer, is in an accessible state of chromatin in most cancers and PDL1 is regulated by a variety of regulatory elements (1).

Several high-throughput techniques have been developed to profile chromatin accessibility, such as Assay for Transposase-accessible Chromatin Sequencing (ATAC-seq) (6), formaldehyde-assisted isolation of regulatory elements (7), DNaseI hypersensitivity coupled with high-throughput sequencing (8, 9) and micrococcal nuclease digestion followed by high-throughput sequencing (10, 11), in which ATAC-seq requires only a small number of cells and becomes a powerful technology with high accuracy and sensitivity to profile genome-wide chromatin accessibility (6). Several databases have stored chromatin accessibility data, such as Cistrome (12), TCGA (https://portal.gdc.cancer.gov/) and ENCODE (13). They have been effective data sources for chromatin accessibility investigation. However, these available resources do not annotate cancer-related CARs. Space (http://fun-science.club/SPACE/) is a web server for linking chromatin accessibility with clinical phenotypes and the immune microenvironment in pan-cancer analysis that effectively helps cancer researchers better understand the immune microenvironment of pan-cancer. However, detailed data on each type of cancer is not provided. Moreover, this database does not have TF binding site information and other related annotation information such as SNPs, expression quantitative trait loci (eQTLs), copy number variation (CNV), single nucleotide variants (SNVs), enhancer and 450K methylation sites.

Here, we developed a comprehensive cancer chromatin accessibility database (CATA, http://www.xiejjlab.bio/cata/), which aims to provide available resources of cancer CARs and to annotate their potential roles in the regulation of genes in cancer type-specific manner. By integrating annotated data from various databases, including TCGA (1), FANTOM (14), 1000 genomes (15), Jaspar (16) and Xena (17), CATA stores CARs and corresponding regulatory annotations across different human tumor samples. CATA also supplies the clinical characteristics for every tumor sample that enables researchers to determine the prognosis prediction value of driver genes by survival analysis. CATA also provides multiple user-friendly functions for data storage, browsing, annotation and analysis. It could be a powerful work platform for mining potential functions of CARs and exploring relevant regulatory patterns about cancer.

## Materials and methods

### The collection of chromatin-accessible regions

We downloaded chromatin accessibility region data (.bed file) from TCGA across 23 cancer types, covering 410 samples (Table 1). These regions were identified from ATAC-seq data according to the processing pipeline of TCGA (1, 18, 19). First, the ATAC-seq data processing and alignment were performed using the PEPATAC pipeline (http://code.databio.org/PEPATAC/). The hg38 genome build used for alignment was obtained from Refgenie (https://github.com/databio/refgenie). Bowtie2 was used to align the ATAC-seq data to the hg38 human reference genome using ‘--very-sensitive - X 2000 –rg -id’ options. Picard (http://broadinstitute.github.io/picard/) was then used to remove duplicates. For each sample, peak calling was performed on the Tn5-corrected single-base insertions using the MACS2 (20) callpeak command with parameters ‘--shift -75 --extsize 150 --nomodel --call-summits --nolambda --keep-dup all -p 0.01’.The peak summits were then extended by 250 bp on either side to a final width of 501 bp. The hg38 blacklist (https://www.encodeproject.org/annotations/ ENCSR636HFF/) was then used to filter and finally remove peaks that extend beyond the ends of chromosomes. For the overlapping peaks in a single sample, the most significant peak is retained, and any peak directly overlapping with the significant peak is eliminated. Finally, each sample has a set of fixed-width peaks. For each cancer, TCGA compiled a ‘cancer type-specific peak set’ containing all of the reproducible peaks observed in an individual cancer type. For the overlapping peaks from different samples, TCGA kept the most significant peak. At last, the ‘Pan-cancer Peak Set’ was obtained from the most significant peak of all the cancer types that could be used for cross-cancer comparison.

**Table 1.** Detailed information on various cancer samples

Cancer type	Shorthand	Sample number
Adrenocortical Cancer	ACC	9
Breast Cancer	BRCA	75
Stomach Cancer	STAD	21
Colon Adenocarcinoma	COAD	41
Kidney Renal Papillary Cell Carcinoma	KIRP	34
Prostate Adenocarcinoma	PRAD	26
Lung Adenocarcinoma	LUAD	22
Esophageal Carcinoma	ESCA	18
Liver Hepatocellular Carcinoma	LIHC	17
Kidney Renal Clear Cell Carcinoma	KIRC	16
Lung Squamous Cell Carcinoma	LUSC	16
Thyroid Carcinoma	THCA	14
Brain Lower Grade Glioma	LGG	13
Skin Cutaneous Melanoma	SKCM	13
Uterine Corpus Endometrial Carcinoma	UCEC	13
Bladder Urothelial Carcinoma	BLCA	10
Head and Neck Squamous Carcinoma	HNSC	9
Pheochromocytoma and Paraganglioma	PCPG	9
Mesothelioma	MESO	7
Cholangiocarcinoma	CHOL	5
Cervical squamous cell carcinoma and endocervical adenocarcinoma	CESC	4
Glioblastoma Multiforme	GBM	9
Testicular Germ Cell Tumors	TGCT	9
Pan-Cancer (all of above cancer types)	PAN	410

### Chromatin accessibility region annotation

CARs were annotated both genetically and epigenetically using BEDTools (21), including common SNPs, risk SNPs, CNV, SNV, motif changes, eQTLs, transcription factors’ binding sites (TFBS), methylation, enhancers and CRISPR/Cas9 target sites. The annotation information is advantageous in discovering the potential function of chromatin accessibility regions. In addition, interactive tables are used to further illustrate the details.

### Enhancer collection

In total, 65 423 enhancers were collected from FANTOM5 (14) and then converted to hg38 genome by LiftOver tool (22) for the annotation.

### TF-related data

TF binding regions were obtained from FIMO (23) prediction with the parameters ‘-verbosity 1 --skip-matched-sequence --thresh 1e-6 --parse- genomic-coord’. Besides, 5 797 266 TFBS were downloaded from the UCSC (22) and converted to the hg38 genome by LiftOver (22).

### CRISPR/Cas9 target sites

CRISPR/Cas9 target (24) can be used in tumor cells to precisely shear genomic loci. CRISPR/Cas9 gRNA sequences target DNA sequences of transcription regions within 200 bp of genomic regions. We downloaded the CRISPR/Cas9 information from UCSC and converted it to HG38 via Liftover (22). We used the CRISPOR tool (25) for prediction to help design, evaluate and clone guidance sequences for the CRISPR/Cas9 system.

### Gene annotation

The ROSE genemapper (26) method was applied in the prediction of CAR-associated genes. The genemapper method based on their distance in the linear genome to identify target genes of regulatory regions. Notably, three strategies, including overlap (genes in the CAR region), proximal (Genes within 50kb of the CAR) and closest (the gene closest to CAR), were adopted to locate CAR-associated genes.

### Common SNPs/linkage disequilibrium SNPs/Risk SNPs

Total 38 063 729 common SNPs were downloaded from dbSNP (27) version 1.50. Linkage disequilibrium (LD) SNPs were calculated using phased genotype information from 1000 genomes project phase 3 (15). Then, a minimum filtered SNP allele frequency of less than 0.05 (MAF) was accepted through VCFTools (28) (v0.1.13). Finally, plink (29) (v1.9) was utilized to calculate SNPs with MAF > 0.05 in LD (r2 = 0.8) for five subgroups (Africa, ad mix America, East Asia, Europe and South Asia). Risk SNPs, genome-wide association studies (GWASs) were collated from GWAS catalog (30) and GWASdb (31) v2.0, which contain SNP insertion/deletion variation annotated in human diseases/traits.

### Motif changes

We collected position weight matrices from TRANSFAC (32) and JASPAR (16) to explain the effect of annotation mutations on motifs. Then, we used the R package at SNP (33) to calculate the binding affinity of mutation to motifs. SNP mutations affect the binding affinity of mutations to the motif and make the binding of the motif change accordingly. The 30-bp region upstream and downstream of SNPs with MAF > 0.05 of 1000 Genomes Project (15) phase3 that located in super-enhancer regions was calculated. Ultimately, we obtained 254 545 586 motif changes.

### TCGA series data

TCGA-related data were obtained from UCSC XENA (17) (http://xena.ucsc.edu/), including methylation data, RNA expression profile data and somatic-mutation-variation, copy-number-variation, clinical information, ATAC-seq raw counts numbers. Besides, methylation and RNA expression profiles were averaged based on the type of cancer.

### EQTL

We downloaded and merged human eQTL data sets from GTEx (34) v5.0, HaploReg (35) and PancanQTL (36). The data of GTExV5.0 and HaploReg mainly included the relationship between eQTL and genes in different tissues. PancanQTL data included relationships between eQTL and genes of different cancers in TCGA (https://tcga-data.nci.nih.gov/tcga). We mapped and annotated eQTL-related SNPs to CARs and provided SNP-regulated genes as potential targets for CARs.

### System design and implementation

CATA is built using MySQL (http://www.mysql.com), running on Linux based Tomcat Web server (http://tomcat.apache.org/). The main framework of CATA was developed based on Java 1 0.8.0 with Springboot and MySQL 5.7.16. The front end was designed and built using Bootstrap v4.1.1 (https://v4.bootcss.com). The network visualization was accomplished through Echarts (37) (https://www.echartsjs.com/). Chromatin visualization is provided by Genomic Visualization Engine (38) (GIVE) (https://zhong-lab-ucsd.github.io/GIVE_homepage/). We recommend a minimum display resolution of 1440 × 900 and using a modern web browser, which supports the HTML5 standard such as Firefox (v90) and Google Chrome (v90) to achieve the best display.

### Pathway analysis

CATA provides 10 choices about pathway databases (KEGG, Reactome, NetPath, WikiPathways, PANTHER, PID, HumanCyc, CTD, SMPDB and INO). According to the following formula:

$$\begin{equation*}{\ }P = 1 - \mathop \sum \limits_{i = 0}^{a - 1} {{\left( {\begin{matrix}
n \cr
i \cr
\end{matrix} } \right)\left( {\begin{matrix}
{t - n} \cr
{z - i} \cr
\end{matrix} } \right)} \over {\left( {\begin{matrix}
t \cr
z \cr
\end{matrix} } \right)}}\end{equation*}$$

where *t* is the number of genes of the entire genome, and *z* is the number of genes of interest, of which a gene is involved in the pathway containing *n* gene. The calculated *P*-value is provided on the result page, along with the relevant genes, as well as the pathway ID (pathway ID can be clicked into the detailed pathway information). The false discovery rate (FDR) method is used to correct for multiple testing. Users can adjust the number of genes required to be enriched and set thresholds of *P*-values or FDRs to control the stringency of analysis.

### Database maintenance

We have a professional database maintenance team that regularly maintains and upgrades the database. At the same time, for the ever-increasing data, we will regularly add corresponding data to the database every year, such as some new cancer open data and new annotation data.

## Results

### Data content

**Figure 1.**
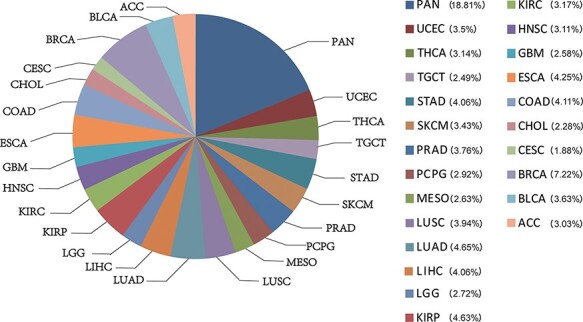
The percentage of chromatin-accessible region for per cancer type.

**Figure 2.**
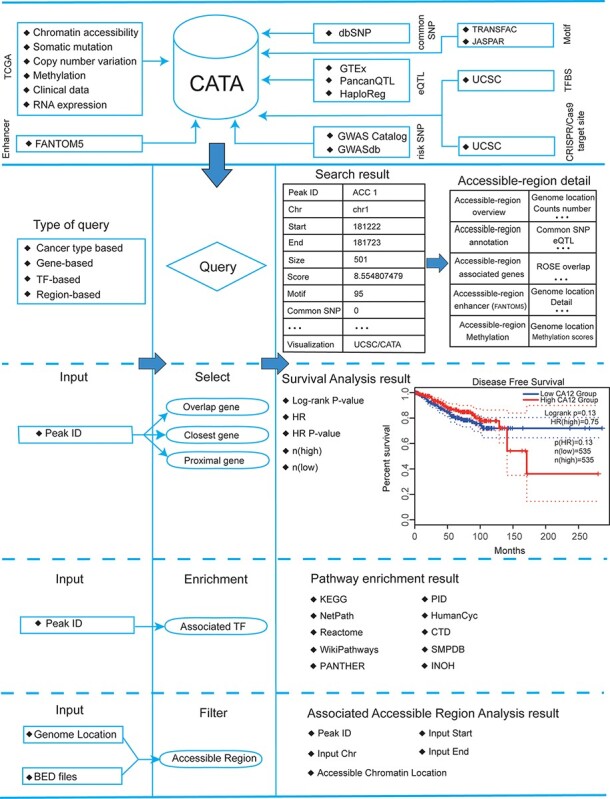
Database content and construction. CATA provides chromatin-accessible regions of cancer-based on TCGA ATAC-seq data. Genetic and epigenetic annotations of accessible regions were collected or calculated including common SNPs, eQTLs, risk SNPs, LD SNPs, TFBS, CNV, SNV, methylation sites and enhancer location. CATA also provides ATAC-seq samples associated with clinical data. CATA integrates multiple functions including storage, search, download, statistics, visualization, browse and analysis.

CATA stores 2 991 163 CARs and corresponding regulatory annotations for 410 tumor samples across 23 cancer types (ACC, BLCA, BRCA, CESC, CHOL, COAD, ESCA, GBM, HNSC, KIRC, KIRP, LGG, LIHC, LUAD, LUSC, MESO, PCPG, PRAD, SKCM, STAD, TGCT, THCA, UCEC) and PAN (Figure 1). Annotated data is integrated from 12 databases in CATA, including TCGA, FUNTOM, 1000 genomes, Jaspar and Xena. The annotated data includes SNPs (38 063 729), risk SNPs (264 514), eQTLs (2 886 133), CNV (9408), SNV (8631), enhancer locations (65 424) and conserved TFBS (5 797 266) (Figure 2 and Table 2).

**Table 2.** Annotate data sources and quantities

DATA types	Numbers	Source
TFBSs conserved	5 797 266	UCSC
CRISPR/Cas9 target sites	22 620 266	UCSC
Risk SNPs	264 514	GWASdb and GWAScatalog
Common SNPs	38 063 729	dbSNP
Motif changes	254 545 586	CATA
Chromatin accessibility region	2 991 163	TCGA
eQTLs number:	2 886 133	GTEx v5.0, HaploReg and PancanQTL
Enhancer	65 424	FUNTOM
Copy-number-variation	9408	XENA
Somatic-mutation-variation	8631	XENA

### User-friendly explorations

**Figure 3.**
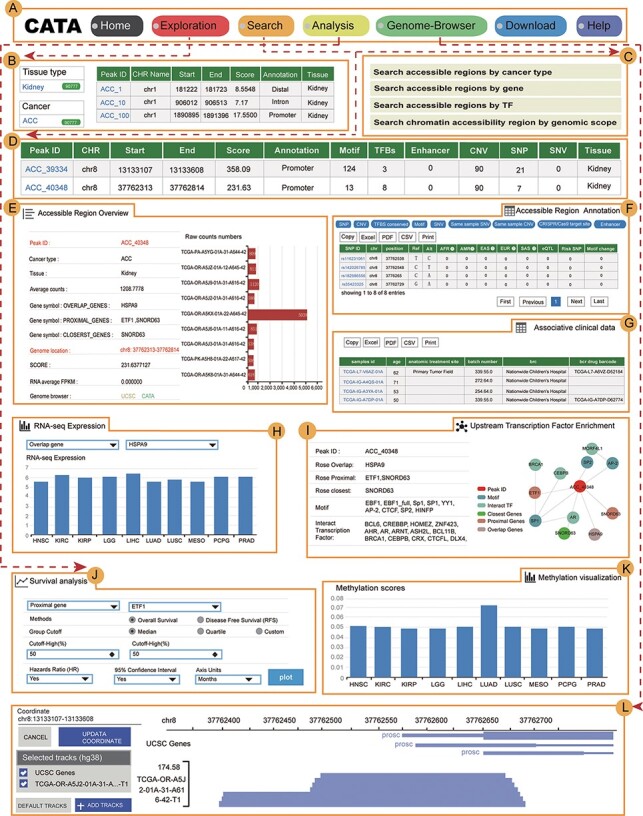
The main function and usage of CATA. (A) The navigation bar of CATA. (B) CATA provides user’s friendly explorations. (C) Users can query using five methods: ‘Search by Cancer type’, ‘Search by gene’, ‘Search by TF’ and ‘Advanced search by genome location’. (D) The display of search results. (E) Overview of chromatin-accessible regions. The *y*-axis is the patient ID provided by TCGA. The *x*-axis is the count number that is the raw read count of ATAC-seq in the Peak ID. (F) Interactive table of chromatin-accessible region, related annotation information. (G) The table of clinical data. (H) The visualization of RNA-expression. (I) Upstream TF enrichment graph. (J) The overall survival and disease-free survival analysis of the interest gene can be presented in the ‘Survival’ region. Meanwhile, genes with the most significant association with patient survival can be identified. (K) The visualization of the methylation level. (L) Personalized genome browser-GIVE.

CATA provides a user-friendly explanation interface to help users navigate quickly and easily (Figure 3A). On the left side of the exploration page, the user has four options to distinguish between different samples (tissue type, cancer type, annotation and chromosome) and use these options to filter the results (Figure 3B). Also, the user can click on ‘Peak ID’ to navigate to the detail page to learn more about CAR information.

### A search interface for retrieving CAR

In the ‘Search’ page, users can get chromatin accessibility data through four strategies, including ‘Search accessible regions by cancer type’ (input cancer type), ‘Search accessible regions by gene’ (input gene of interest, cancer type and strategies), ‘Search accessible regions by TF’ (input TF name of interest and cancer type) and ‘Search chromatin accessibility region by genomic scope’ (input cancer type and genomic position) (Figure 3C). In search accessible regions by cancer type, cancer type was used as an input. The output table first displayed the brief annotation information of CARs ID (Figure 3B). This table consists of Peak ID, genome location, start, end, score of CARs, annotation of region, numbers of motif, numbers of TFBS, enhancer numbers, CNV, SNP, SNV and tissue type. The word ‘TFBS’ is used to define a particular sequence (genetic or artificial), which is a place where factors combine. The word ‘motif’ is used for binding specific genetic descriptions, which are obtained by aggregating information from a series of sites. The user gets more annotations according to the interested Peak ID. The usage method is same as the usage of ‘Search accessible regions by gene’ and ‘Search accessible regions by gene. In searching accessible regions by gene, users input interest gene in ‘Gene Symbol’, cancer type in ‘Cancer Type’ and chose one of ‘Strategies/Algorithm’. The ‘Example’ option is an example that was provided by CATA. After click on the ‘search’, the brief information of interest gene on the search results is displayed in a table on the result page (Figure 3D). After clicked in Peak ID, CATA provides a preview of CARs (Figure 3E), including raw count numbers, presented by bar plots and some summary information (overlap genes, proximal genes, closest genes, genome location, score of CARs, RNA average FPKM) about the accessible region of interest gene inaccessible region overview. The *y*-axis is the patient ID provided by TCGA in the plot. The *x*-axis is the count number that is the raw read count of ATAC-seq in the Peak ID. Users can search for interest gene annotation through the UCSC and CATA portals.

In accessible region annotation, annotation CARs information of CARs regions is provided in tabular form, including SNP, motif, CNV, SNV, TFBS, etc. (Figure 3F). Users can also download related annotation information. Users can choose three strategies to get gene expression levels in a variety of tumors, including closest gene, overlap gene and proximal gene of ‘Peak ID’ (Figure 3H). Users also have the option to download raw clinical data for analysis (Figure 3G) or to perform survival analysis online (Figure 3J). What CATA provides in the survival analysis section is GEPIA’s analysis strategy, linked to GEPIA’s online survival analysis (39). CATA also provides methylation visualization in 23 cancer types (Figure 3K). In upstream TF enrichment, interest gene binding TF and interacted gene can be obtained directly (Figure 3I).

Meanwhile, users can only input CAR ID to complete pathway enrichment online in CATA. CATA also supports the ‘Threshold’ option, allowing users to set different thresholds to ensure that the pathway enrichment for each user is highly accurate and suitable. For instance, we input in ‘Peak ID’ and chose related databases. The threshold is set to whatever users want. CATA will provide pathway enrichment of related genes. In addition, users can input the genome location to analyze the chromatin accessibility of the region. Users also upload files in the ‘.bed’ format to analyze chromatin accessibility. CATA will then provide summary information (CAR ID, genomic location and brief annotation information) that correlates with the data uploaded by the user. In Genome-Browser, CATA implements CAR visualization using GIVE (Figure 3L). Users can select kinds of cases and tumor types whatever users want to analyze. In the end, users can download gene annotation and associated TF on the ‘Download’ page. CATA provided a download of gene annotation and associated TF files in the ‘.txt’ format for each sample.

### Personalized genome browser and data visualization

CATA deploys genome browser GIVE to visualize the CARs (Figure 3L). We provide 23 types of cancer and a total of 796 bigwig files for visualization of tissue type-specific CARs. Users can enter a region in the navigation bar and load the corresponding track for visualization. We divided all the samples into 23 groups in detail and named the samples according to the TCGA-patient ID.

### Online analysis tools and data download

CATA offers two analytical tools, including (i) pathway downstream analysis, in which users can identify TFs on the CAR region and further mine upstream regulatory pathways enriched by these TFs. Users only need to input the PEAK-ID of interest to perform cell pathway analysis (ii). Associated Accessible Region analysis, in which users can upload one or more genomic locations to our web. CATA also supports the uploading of standard bed files. CATA will then provide summary information (CAR ID, genomic location and brief annotation information) that correlates with the data uploaded by the user.

Besides online analysis, CATA also friendly provides data downloads. Both CARs data and the information about the target gene and the TFBS located in the corresponding region are provided.

### Case studies

**Figure 4.**
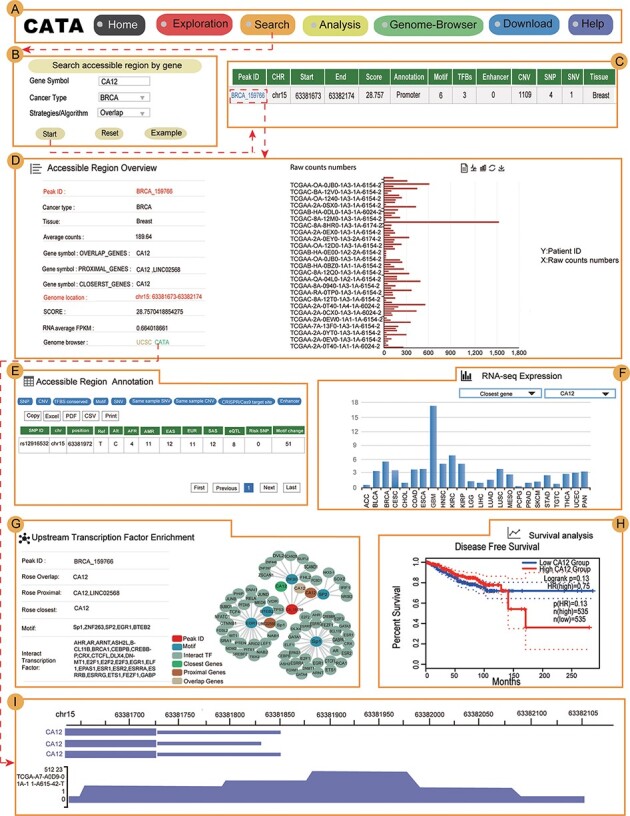
Validation results associated with CA12 in breast cancer. (A) The navigation bar of CATA. (B) Input and parameters of ‘Search accessible regions by gene’. (C) The brief annotation information about the detailed genetic information in chromatin-accessible regions of CA12, including SNP, motif, CNV, SNV, TFBS, etc. The score is a score of chromatin accessibility provided by TCGA. The higher the score, the more open the chromatin. (D) In accessible region overview, annotation CAR information of CA12 CAR regions including raw count numbers, presented by bar plots and some summary information (overlap genes, proximal genes, closest genes, genome location, score of chromatin-accessible regions, RNA average FPKM) about the accessible region of interest gene. The *y*-axis is the patient ID provided by TCGA. The *x*-axis is the count number that is the raw read count of ATAC-seq in the Peak ID. (E) In accessible region annotation, annotation CAR information of CAR regions are provided in tabular form, including SNP, motif, CNV, SNV, TFBS, etc. (F) The visualization of RNA expression of CA12. (G) Upstream TF enrichment of the CA12 graph. (H) The disease-free survival analysis of CA12. (I) Visualization of the CA12 chromatin-accessible region.

Users can use a user-friendly explanations interface to quickly and easily find their interested aspects (Figure 4A) in CATA. To provide examples about how to use CATA to identify CARs in cancer, Carbonic anhydrase XII (CA12) and FOXA1 were used as inputs on the website.

CA12 is a transmembrane, extracellular enzyme and member of a family of zinc metalloenzymes that catalyze the reversible hydration of CO_2_ to form bicarbonate, regulating the microenvironment acidity and tumor malignant phenotype. In searching accessible regions by gene, we input ‘CA12’ in ‘Gene Symbol’, ‘BRCA’ in ‘Cancer Type’ and ‘Overlap’ in ‘Strategies/Algorithm’. ‘Example’ option is the example that was provided by CATA (Figure 4B). After click on ‘Start’, the brief information of CA12 on the search results is displayed in a table on the result page (Figure 4C). This table consists of Peak ID, genome location, start, end, score of CARs, annotation of region, numbers of motif, numbers of TFBS, enhancer numbers, CNV, SNP, SNV and tissue type. We choose one of the Peak IDs, for example ‘BRCA_159766’. After clicked in ‘BRCA_159766’, some summary information of accessible region of CA12 was revealed, including including raw count numbers presented by bar plots, overlap genes, proximal genes, closest genes, genome location, score of chromatin-accessible regions and RNA average FPKM (Figure 4D). We can search for CA12 CARs through the UCSC and CATA portals.

In ‘accessible region annotation’, we can get annotation of the accessible region of CA12, including SNP, motif, CNV, SNV, TFBS, etc. (Figure 4E). There are three strategies to analyze gene expression levels in a variety of tumors, including closest gene, overlap gene and proximal gene of CA12 (Figure 4F). We found RNA-seq expression of CA12 in breast cancer was higher than the majority of cancers. Univariate analysis was conducted to explore the relation of CA12 to established prognostic factors and survival. Indeed, P.H. Watson reported that CA12 is a marker of good prognosis in invasive breast carcinoma (40). Expression of CA12 was all significantly related to disease-free survival (Figure 4H), which could be identified by inputting CA12 in CATA (Search accessible regions by gene). Meanwhile, the CA12 gene is under primary transcriptional up-regulation by the estrogen-occupied ESR1, and that this regulation in breast cancer cells is mediated by ER action through a distal enhancer that we have herein characterized (41). GATA3 influences the expression of CA12 in mediating ESR1 binding by shaping enhancer accessibility (42). After searching CA12 in ‘Search accessible regions by gene’, we found the interacting TFs GATA3 and ESR1 by scanning the CARs of CA12 in upstream TF enrichment (Figure 4G). Meanwhile, CATA provided many TFs that interacted with CA12, including BRCA, SOX2, E2F1 and MYC. Indeed, these TFs were reported to be associated with chromatin accessible (43–46), for example, SOX_2_. TF SOX_2_ is a part of the core regulatory network that determines chromatin accessibility, epigenetic modification and gene expression patterns in esophageal squamous cell carcinoma cell lines (45). All this information provides new opinions for users to study breast cancer. In Genome-Browser, CATA implements CARs of CA12 visualization using GIVE (Figure 4I).

In search region, CATA can also search CARs by inputting certain transcriptional factor (Supplementary Figure S1A). For example, in ‘Search accessible regions by TF’, we input ‘FOXA1’ in ‘Motif’, ‘Cancer Type’ in ‘PRAD’ and started (Supplementary Figure S1B). Then, CARs that FOXA1 possiblly binds were shown in the page. We choose peak ID ‘PRAD_103425’ to get specific information of interested region and its downstream genes, such as clinical data, methylation levels, RNA expression levels, survival analysis and other upstream TFs (Supplementary Figure S1C). In the ‘Upstream Transcription Factor Enrichment’ section, we can get the TFs bound to the open region bound by FOXA1, such as ESR1, GATA3, AR and so on (Supplementary Figure S1D). FOXA1 is a driver factor in the incidence and progression of prostate cancer (47–49). As a pioneer factor, FOXA1 improves chromatin accessibility for subsequent binding to lineage-specific TFs such as AR in prostate tissue. The chromatin recruited by FOXA1 promotes lost DNA methylation and existing histone methylation, especially H3K4me1 and H3K4me2 modifications (50, 51). Therefore, FOXA1 may affect the binding of AR in this area by affecting the accessibility of chromatin in this area. The following usage is shown in the above section. In the analysis section, we can upload the peak IDs we are interested in to conduct channel enrichment analysis. For example, after we clicked on ‘Example’, clicked ‘Start’ and we got pathway enrichment of upstream TFs. Also, we inquire whether some genomic regions are accessibile for chromatin, we could input genomic scope or upload ‘bed’ files.

## Discussion

Chromatin accessibility plays a critical role in tumorigenesis. In cancer cells, CARs are frequently bound by TFs and contain much information about genetics. Some database has already stored chromatin accessibility data, such as Cistrome, TCGA and ENCODE. They have become useful data sources for studying chromatin accessibility. Compared with Cistrome and ENCODE, CATA is a chromatin accessibility database that focuses on cancer and provides extensive opening region annotation information. Compared with other existing databases, CATA not only stores 2 991 163 CARs from 23 cancer types but also provides comprehensive annotations about these regions, including common SNPs, risk SNPs, CNVs, somatic mutations, motif changes, eQTLs, TF binding regions, methylation, enhancer location and CRISPR/Cas9 target loci. Moreover, CATA supports cancer survival analysis of CAR-associated genes that helps researchers to identify driver genes.

CATA database mainly includes five user-friendly characteristics: (I) CATA provides four strategies, including ‘Searchaccessible regions by cancer type’ (input cancer type), ‘Search accessible regions by gene’ (input gene of interest, cancer type and strategies), ‘Search accessible regions by TF’ (input TF name of interest and cancer type) and ‘Search chromatin accessibility region by genomic scope’ (input cancer type and genomic position). (II) CATA has a more user-friendly ‘Explorations’ page. (III) CATA provides two analytical tools, including pathway downstream analysis and associated accessible region analysis. (IV) CATA supports data download of 23 types of cancers. (V) CATA provides detailed help documentation to quickly use and understand the database. In the future versions, we will provide relevant ChIP-seq data, cancer single-cell ATAC-seq data and practical analysis tools. This will lead to a better exploration of tumorigenesis mechanisms and cancer markers.

In summary, CATA is a novel chromatin accessibility database for cancer that provides a general collection of cancer CARs. Especially, CATA provides the most extensive cancer chromatin accessibility annotation. CATA provides an easy-to-use database platform for researchers to explore cancer CARs and detailed features. Our effort to establish this database was prompted by the great need of researchers to a comprehensive dataset of cancer-related CARs for their related genomic location, target genes, TFBS, mutation, methylation, functions and survival analysis. We expect that CATA will help researchers to understand cancer more comprehensively by providing this information in an integrative manner.

## Supplementary Material

baab085_Supp

## Data Availability

Publicly available datasets were analyzed in this study. This data can be found here: http://www.xiejjlab.bio/cata/.
