# Supplementary material for: CATA: a comprehensive chromatin accessibility database for cancer
Source: Database (Oxford). 2022 Jan 17;2022:baab085. doi: 10.1093/database/baab085 (PMC9246274; doi:10.1093/database/baab085)
Supplement: baab085_Supp [file baab085_supp.zip › Supplementary Figure Legend 1.docx]

Supplementary Figure 1. Validation results associated with FOXA1 in prostate cancer. (A) The navigation bar of CATA. (B) Input and parameters of ‘Search accessible regions by TF’. (C) The brief annotation information about the detailed genetic information in chromatin-accessible regions bound by FOXA1, including SNP, motif, CNV, SNV, TFBS, etc. The score is a score of chromatin accessibility provided by TCGA. (D) Upstream transcription factors predicted by FOXA1 binding region.
