# Supplementary figures and images for: CATA: a comprehensive chromatin accessibility database for cancer
Source: Database (Oxford). 2022 Jan 17;2022:baab085. doi: 10.1093/database/baab085 (PMC9246274; doi:10.1093/database/baab085)

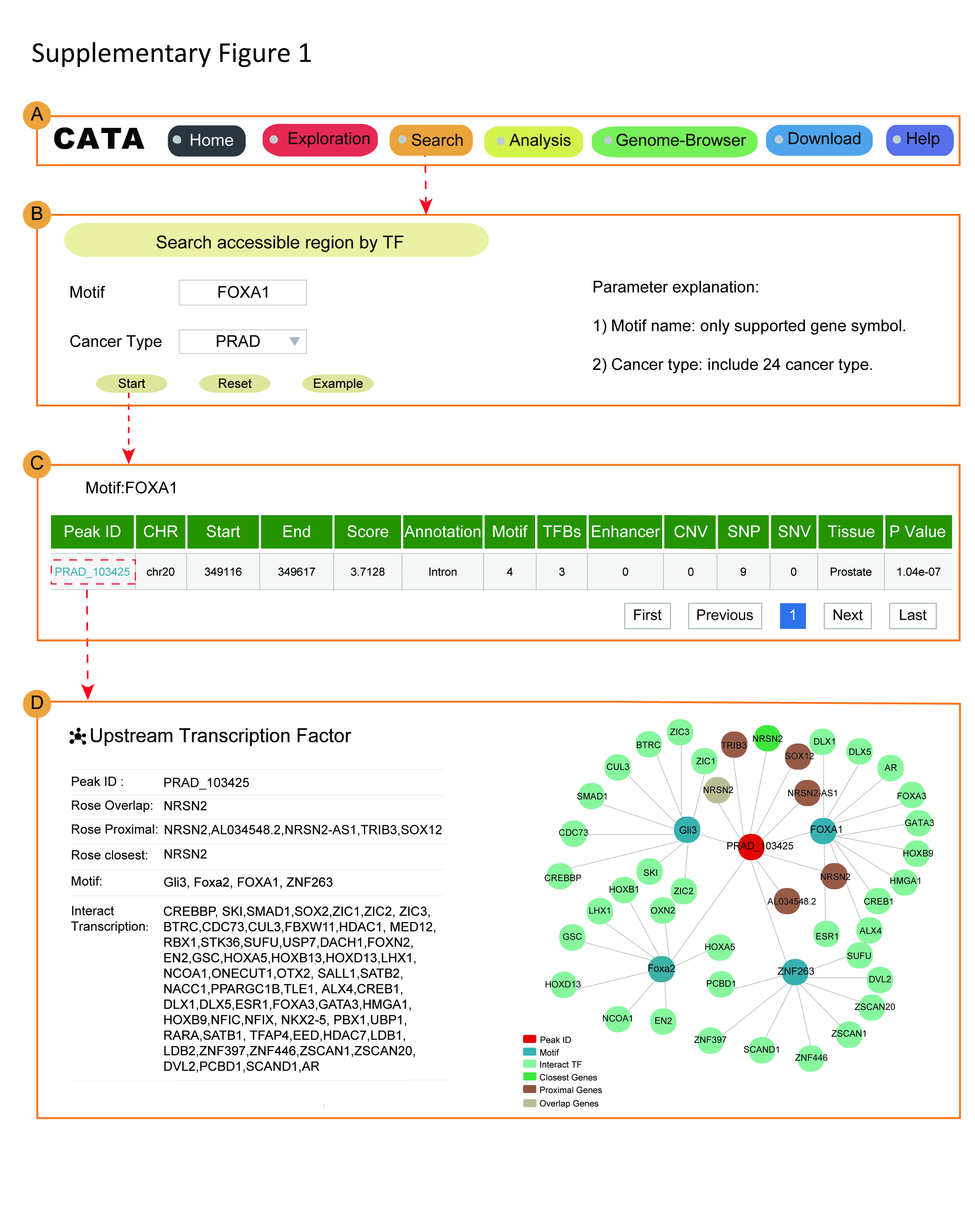

Supplement: baab085_Supp [file baab085_supp.zip › Supplementary Figure 1(1).tif]
